# Is Vitamin D3 a Worthy Supplement Protecting against Secondary Infections in Dogs with Atopic Dermatitis?

**DOI:** 10.3390/pathogens12010145

**Published:** 2023-01-15

**Authors:** Dorota Chrobak-Chmiel, Anna Golke, Ewelina Kwiecień, Małgorzata J. Biegańska, Kourou Dembele, Małgorzata Dziekiewicz-Mrugasiewicz, Michał Czopowicz, Magdalena Kizerwetter-Świda, Magdalena Rzewuska

**Affiliations:** 1 Department of Preclinical Sciences, Institute of Veterinary Medicine, Warsaw University of Life Sciences, Ciszewskiego 8, 02-786 Warsaw, Poland; 2 Department of Small Animal Diseases and Clinic, Institute of Veterinary Medicine, Warsaw University of Life Sciences, Nowoursynowska 159c, 02-776 Warsaw, Poland; 3 Department of Large Animal Diseases with Clinic, Faculty of Veterinary Medicine, Warsaw University of Life Sciences, Nowoursynowska 100, 02-797 Warsaw, Poland; 4 Division of Veterinary Epidemiology and Economics, Institute of Veterinary Medicine, Warsaw University of Life Sciences, Nowoursynowska 159c, 02-776 Warsaw, Poland

**Keywords:** canine atopic dermatitis, vitamin D3, antimicrobial peptides, *Staphylococcus pseudintermedius*, *Malassezia pachydermatis*

## Abstract

Canine atopic dermatitis (CAD) is a common, chronic, inflammatory skin disease in dogs worldwide. This disease often predisposes for secondary organisms overgrowth and skin infections with pathogens, such as *Staphylococcus pseudintermedius* and *Malassezia pachydermatis*. Unfortunately, the causes of this disease in both humans and animals are not fully understood; therefore, the only possible option is a lifelong, symptomatic treatment. The management of CAD is mainly based on limiting contact with allergens and antipruritic therapy, most often with glucocorticoids and antihistamines. A serious problem in this situation is the fact, that long-term administration of glucocorticoids leads to side effects like polyuria, alopecia, increased susceptibility to infection, muscle atrophy, and many others. For this reason, great emphasis is placed on the development of replacement and supportive therapies. It is a well-documented fact that reduced concentrations of serum vitamin D3 contribute to the severity of atopic dermatitis symptoms in humans. Moreover, unlike the most commonly used therapeutic methods, of which the main goal is to ameliorate inflammation and pruritus, namely the symptoms of AD, vitamin D3 supplementation affects some underlying factors of this disease. Therefore, in this review, we summarize the current state of knowledge regarding the role of vitamin D3 in CAD, its protective effect against secondary bacterial and fungal infections, and the potential of its supplementation in dogs.

## 1. Canine Atopic Dermatitis

Canine atopic dermatitis (CAD) is one of the most common inflammatory and pruritic skin diseases in dogs worldwide. CAD is a multifactorial disease that results from complex interactions between genetic and environmental factors. It is associated with the production of antibodies from the immunoglobulin E (IgE) class against multiple environmental allergens, such as pollens, mites, molds, food allergens, and some microorganisms, such as *Malassezia* spp. or staphylococci [1]. However, the pathogenesis is complicated and only partly understood. Genetic abnormalities, altered immune response to cutaneous inflammation, and a skin barrier defect contribute to the development of the disease. It is known that the primary defect in the epidermal barrier in atopic dogs, such as decreased ceramide levels or filaggrin concentrations, facilitates the penetration of allergens through the epidermis, and this leads to the over-stimulation of the local innate and adaptive immunity [2,3]. Skin barrier function is further impaired by inflammation. Moreover, the weaker the skin barrier, the higher the predisposition to allergic sensitization. Allergens absorbed through the skin promote allergen-specific IgE production which binds to tissue mast cells and basophils. After re-exposure to the antigen, mast cells degranulate releasing histamine, proteolytic enzymes, and bradykinins. This all leads to the development of skin inflammation manifesting itself with erythema (redness), edema (swelling), and pruritus (itching) [4]. Furthermore, various allergens stimulate the synthesis of interleukin (IL)-4 by dendritic cells, leading to a polarization towards the lymphocyte T type 2 (Th2)-associated response. This is further exacerbated by the lymphopoietin (similar to IL-7) produced by keratinocytes. Lymphocyte T type 1 (Th1) and Th2 populations differ in the profile of synthesized cytokines. Th1, by producing IFN-γ, IL-2, IL-12, and TNF-α supports the cellular response, whereas Th2 produces IL-4, IL-5, IL-6, IL-13, and IL-31 which in turn stimulate a humoral response [5,6]. IL-4 is believed to play a principal role in the development of atopy. It enhances the proliferation of B lymphocytes and stimulates them to produce IgE. Moreover, IL-4 dysregulates the production of microRNA in keratinocytes, and that leads to enhanced inflammation, angiogenesis, lymphangiogenesis, and apoptosis of epidermal keratinocytes [7,8,9,10]. Additionally, it was proved that canine IL-31 induces pruritus in atopic dogs, leading to epidermal damage through scratching which promotes secondary microbial infections [6].

It is estimated that CAD affects 3–15% of the dog population worldwide [11]. As the diagnosis of CAD is difficult, these numbers are likely to be underestimated [11,12]. The following dog breeds appear to be particularly predisposed to the development of CAD: Boxer, West Highland White Terrier, French Bulldog, Bullterrier, American Cocker Spaniel, English Springer Spaniel, Poodle, Chinese Shar-Pei, Dachshund, Collie, Miniature Schnauzer, Lhasa Apso, Pug, and Rhodesian Ridgeback [3]. The predilection is suspected to be related to the genetic characteristics of these breeds. 

Clinical signs usually emerge for the first time in early adulthood (1–3 years of age), and the most affected body regions are the head, distal limbs (carpal and tarsal regions, paws, mainly digits, claws, and interdigital spaces), ventral part of the abdominal region, perineum, and ventral tail [1]. The main clinical signs are erythema and pruritus, which are exacerbated by self-trauma due to scratching and biting, as well as by secondary infections. Thus, an array of secondary skin lesions such as excoriations, self-induced alopecia, papules, pustules, crusts, erosions, epidermal hyperplasia, hyperpigmentation, and lichenification are often observed [13].

The definitive diagnosis of CAD relies on the combined assessment of medical history and clinical signs, including a characteristic pattern of lesions, and positive result of allergy tests, supported by concomitant elimination of other possible pruritic dermatoses caused by primary parasitic, bacterial, and fungal skin infections or food allergies [1,14]. Allergy testing, either based on serological assays or intradermal skin tests with various allergens, plays only an auxiliary role due to low diagnostic specificity [13]. Dogs often become sensitized to multiple environmental allergens, and therefore they test positive in allergy tests despite the lack of signs of CAD. On the other hand, dogs with clinical signs indicative of CAD may test negative in allergy tests; however, in such a case, the disease is referred to as atopic-like dermatitis [13].

## 2. Treatment of CAD

CAD is a chronic disease, and early intervention is needed to control the clinical signs. A complete clinical and laboratory examination is important in recognizing the main factors that influence disease development and progression and subsequently in determining the best treatment. Unfortunately, the primary cause of CAD remains unknown, and the disease remains incurable. Therefore, the only possible option is a lifelong symptomatic treatment [15]. Once a dog is determined to have atopy, allergy testing may be performed by a veterinary dermatologist to establish the specific allergens that are triggering the allergy issues. Moreover, in order to protect the epidermal barrier and to make the affected dogs feel more comfortable, anti-pruritus treatment is often used. Routine therapeutic protocols include the use of glucocorticoids, antihistamines, ciclosporin, oclacitinib, monoclonal antibody Lokivetmab, omega-6/omega-3 fatty acid supplements, allergen-specific immunotherapy (“hyposensitization”), and topical antipruritic agents. The aforementioned drugs/supplements are used alone or in combinations [16]. Additionally, the affected dog should be placed on a permanent program of flea control.

Glucocorticoids and antihistamines are used to reduce the pruritus [17,18,19]. Glucocorticoids might be applied topically or systemically. Due to the fact that they target different cells expressing glucocorticoid receptors, they exhibit a strong antipruritic effect, and their anti-itch properties probably occur secondarily to the reduction of cutaneous inflammation. However, glucocorticoids may have both short- and long-term side effects that cause different problems in dogs. Adverse effects of glucocorticoids commonly result from the long-term use of supraphysiologic doses to control inflammatory or immunologic disorders. Long-term administration may lead to polyuria, polydipsia, bilaterally symmetric alopecia, increased susceptibility to infection, muscle atrophy, and redistribution of body fat [20]. Due to the risk and side effects of glucocorticoid therapy, antihistamines are often used by veterinarians to avoid or reduce the necessary doses of glucocorticoids [16].

Antihistamines are used to reduce histamine release from mast cells. They are a good option to consider, but usually as an adjunctive treatment. They are often used simultaneously with antibiotics and antifungals throughout the life of atopic dogs. Unfortunately, the responses to antihistamines in atopic dogs are unpredictable, thus, the efficacy of antihistamines remains unreliable [16,19].

Another therapeutic option used to control the disease is allergen-specific immunotherapy. This treatment is based on the results of intradermal skin testing or serological allergy testing (blood tests), or a combination of both. Although this method has been well established, not all dogs show the same response to treatment [21].

Oclacitinib is an oral medication used to decrease pruritus. It is a selective inhibitor of janus kinase 1, which is involved in the signaling pathways of the receptors for IL-2, IL-4, IL-6, IL-13, and IL-31, thus it blocks the Th2 pathway [22]. It was proven that their efficacy is comparable with the efficacy of glucocorticoids [23]. The most commonly reported side effects include gastrointestinal problems (such as vomiting, diarrhea, and decreased appetite) and lethargy [24,25].

Since 2017, the monoclonal antibody Lokivetmab (Cytopoint) has been used to reduce itching and skin lesions in atopic dogs [26]. This monoclonal antibody targets IL-31 and inactivates it, which leads to the reduction of itching. This therapy is effective in CAD, except for otitis externa which seems to be resistant to Lokivetmab and only glucocorticoids sufficiently reduce inflammation of the ear canal. Moreover, side effects of Lokivetmab have also been described, and they include drowsiness, vomiting, diarrhea, lack of appetite, pain at the injection site, dermatitis, and pruritus [27,28].

## 3. Secondary Infections in Atopic Dogs

The skin microbiome includes various microorganisms inhabiting the skin. Commensal microorganisms present on a skin surface protect the skin against pathogenic invasion by, for instance, competing with pathogenic microbes for nutrients, they also interact with the innate and adaptive immune system. They may enhance innate immunity and limit pathogen invasion by inducing specialized T lymphocytes to migrate to the epidermis, which occurs in coordination with dendritic cells residing in the skin. Cutaneous dysbiosis is defined as imbalances in the composition of microbial populations which are linked to the development of chronic inflammatory and allergic diseases [14]. It was proven that the composition of skin microbiota is less diverse in atopic dogs than in healthy dogs. The most prevalent bacteria residing on atopic canine skin are staphylococci, mainly *Staphylococcus pseudintermedius* and *Staphylococcus coagulans* [29,30,31]. The intensity of staphylococcal infection correlates with disease severity [32,33]. Most probably, it is due to the fact that peptidoglycan, through toll-like receptor type 2 (TLR2), strongly stimulates the production of thymic stromal lymphopoietin (TSLP) which facilitates Th2 response [34]. Microorganisms present in large numbers on the atopic skin stimulate the release of pruritogenic and inflammatory cytokines from skin cells [2,14]. Pruritus often leads to secondary skin damage that facilitates infections caused either by bacteria or yeasts. Secondary microbial overgrowth or infection are common causes of increased pruritus in already itchy dogs. Thus, the whole process forms a kind of closed circle. Dogs with atopic dermatitis are predisposed to recurrent staphylococcal and *Malassezia pachydermatis* infections in the skin and ears. Skin infections are considered the most prevalent complications of CAD [2,35,36,37]. These secondary infections are often associated with poor control of the disease. According to Favrot (2015) bacterial infections were present in 66% of dogs with CAD, *Malassezia* spp. infection in 50% of dogs, and otitis externa in 33% of dogs with CAD [13]. The staphylococcal mechanisms of the epidermal barrier disruption are still under investigation. However, the reduction of bacterial load on a skin surface evidently reduces the course of the disease and normalizes the epidermal barrier [35]. Moreover, *Malassezia*-associated dermatitis and otitis externa are common clinical problems and are often exacerbated in conjunction with atopic flares. Atopic dogs have developed an IgE hypersensitivity also to *Malassezia* spp. [38].

## 4. The Role of Antimicrobial Peptides in Innate Immunity

Recently, it has been shown that altered epidermal barrier integrity is the major factor involved in the pathogenesis and predisposition to CAD. The high frequency of staphylococcal and *Malassezia pachydermatis* infections in canine atopic skin suggests that the skin of atopic dogs has a defective innate immune response [39]. The outer layer of the skin, the epidermis, serves as a physical barrier against pathogens. In response to the breaching of the epidermal barrier, the effectors of innate immunity constitute the first line of defense against invading pathogens. The most important factors of the skin’s innate immune system include phagocytic cells, such as macrophages, neutrophils, dendritic cells, natural killer (NK) cells, mast cells, basophils, and eosinophils [40]. Additionally, antimicrobial peptides (AMPs) are known to play a crucial role in cutaneous innate immunity [41,42]. These small, protein molecules act as endogenous antimicrobials against bacteria, fungi, protozoa, and viruses. The most studied AMPs in canine epithelial cells of the skin include β-defensins (BDs) and cathelicidin (K9CATH) [43,44]. In different cell types, the expression of AMPs may be either constitutive (neutrophils), or inducible (keratinocytes) by stimuli such as inflammation, a mechanical breach in the skin integrity, or the presence of microorganisms [43].

Defensins are small, cationic peptides with broad-spectrum antimicrobial activity. They protect the host organism against pathogens by serving as endogenous antibiotics, or by signaling to promote chemotaxis. They also play a role in the wound healing process and in other intercellular communication activities [45]. They are responsible for the membrane disruption in Gram-negative bacteria, while in Gram-positive they contribute to the inhibition of cell wall synthesis [46]. Defensins are expressed by certain phagocytic leukocytes and epithelial cells [47]. Genome analysis allowed for recognizing 43 members of the canine β-defensin gene family [47]. The major β-defensins expressed in canine skin are canine β-defensin-1 (cBD-1) and canine β-defensin-103 (cBD-103). Whereas the other antimicrobial peptides, cathelicidins, protect the skin through direct antimicrobial activity or by the initiation of a host response resulting in cytokine release, inflammation, and angiogenesis. They are multifunctional modulators of innate immune responses, synergistically enhancing the IL-1β-induced production of cytokines (IL-6, IL-10) and chemokines such as macrophage chemoattractant proteins (MCP-1, MCP-3) [48]. It was shown that in healthy skin, keratinocytes express low amounts of cathelicidin, whereas on the course of infection or barrier disruption, the expression of this antimicrobial peptide was significantly increased [49]. It was observed that in patients suffering from atopic dermatitis, the process of AMPs induction was highly reduced in pathologically changed skin [50]. Other studies, concerning the participation of AMPs in the pathogenesis of CAD, showed that various AMPs in lesional and non-lesional canine atopic skin were increased. Increased mRNA expression was observed for cBD-1, cBD-122, cBD3-like, and canine cathelicidin. Only mRNA expression of cBD-103 was decreased in naturally affected atopic dogs [39]. The authors suggest that increased expression of cBD-1 could be related to skin infections, as it was proven that microorganisms can stimulate the expression of human β-defensin-1 (hBD-1) [51]. Moreover, increased levels of cBD-1 may also be upregulated by TNF-α, since higher levels of this cytokine were detected in the lesional skin of atopic dogs [52,53]. Antibacterial and antifungal activity of cBD-103 is still being investigated; however, it is believed that decreased level of this peptide could be linked to increased *S. pseudintermedius* and *M. pachydermatis* infection of the canine skin [39,54]. In atopic dogs higher levels of IL-4 and IL-13 have been detected and both cytokines may influence the expression of cBD-103 leading to the downregulation or defective upregulation of this β-defensin. Such deficiency in cBD-103 may contribute to the higher susceptibility to bacterial skin infections in atopic dogs [50].

The molecular regulation of AMPs transcription is poorly understood. Studies on the influence of bacterial infections on the changes in AMPs expression still do not explain these mechanisms. However, the results obtained by Wang et al. (2004) shed new light on this issue [55]. They discovered that the hormonal form of vitamin D3, 1,25-dihydroxyvitamin D3 (calcitriol) [1,25 (OH)2D], directly regulates antimicrobial peptide gene expression, revealing its potential in the treatment of infections.

## 5. Vitamin D3 and Its Emerging Impact on Innate Immunity

Vitamin D has received a lot of attention since the discovery that vitamin D receptors (VDRs) are abundant in most cells in the body and the detection of enzymes involved in the synthesis of the active form of vitamin D, namely 1,25-dihydroxy vitamin D [1,25(OH) 2D] in non-renal sites like skin [56]. Vitamin D3 is a highly potent steroid hormone that maintains calcium homeostasis. There are two main sources of vitamin D3: exogenous by dietary supplementation and endogenous production in the skin stimulated by exposure to sunlight [57]. 

**Figure 1:**
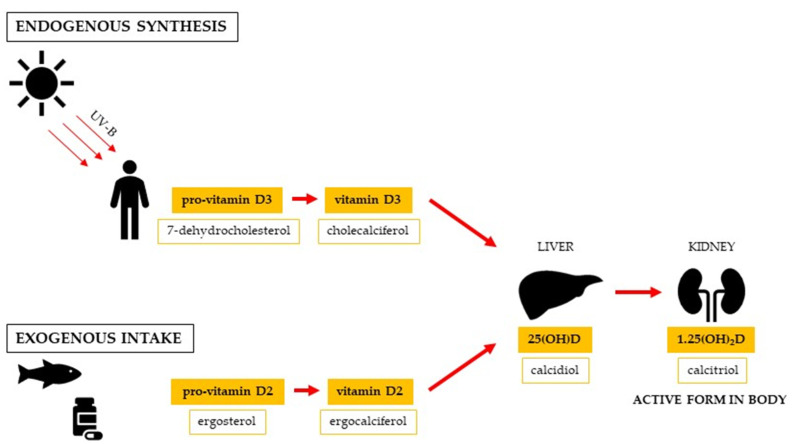
The metabolism of vitamin D in the body.

Two major forms of this vitamin are vitamin D2 (ergocalciferol) and vitamin D3 (cholecalciferol). The skin is not able to synthesize vitamin D2, whereas cholecalciferol can be produced in the skin of most mammals from the pro-vitamin D3 (7-dehydrocholesterol) via activation by ultraviolet B (UVB) light (Figure 1). Dogs appear to have a lower capability to produce cholecalciferol in the skin compared to other mammals [58], which results in the relatively higher dietary requirement for vitamin D. Calcitriol, an active vitamin D metabolite, mediates its biological effects by binding VDR located in the nuclei of target cells. VDR is expressed by many cells, including keratinocytes. This suggests its potential role beyond the bone and calcium metabolism [59]. It was proven that vitamin D3 deficiency leads to a higher risk of bacterial and viral infections [60]. Literature data indicate that vitamin D3 serves as an innate and adaptive immunity regulator [61]. Calcitriol induces the transcription of genes encoding membrane-bound and cytoplasmic pattern recognition receptors (PRRs), such as TLR4, TLR2, and NOD-like receptor 2 (NOD2) [62,63,64]. Vitamin D signaling also activates cytokine production, including interleukin 1β (IL1β) and IL8/CXCL8, during infection [65]. It is a very important mechanism that links innate and adaptive immunity. Additionally, there is a connection between vitamin D3 and AMPs expression in keratinocytes. VDR has been found in the promoter region of the cathelicidin gene [55,66]. Constitutive expression of human cathelicidin (hCAP18 or LL-37) is very low in keratinocytes; however, treatment with the biologically active form of vitamin D,1,25 (OH)2 (vitamin D3) induces LL-37 mRNA expression 100-fold in comparison to unstimulated control [67]. The involvement of cathelicidin in wound healing and skin diseases, such as CAD may create new opportunities for the use of vitamin D3 in dermatology [68].

Furthermore, the expression of LL-37 in monocytes and macrophages has been shown to be induced by vitamin D3, thus leading to enhanced intracellular killing [69]. In keratinocytes, 1,25(OH)2D increases TLR2/1 and LL-37 expression, leading to an increased antimicrobial activity against *Staphylococcus aureus* [67,70].

## 6. Vitamin D3 Levels and Its Presumptive Impact on CAD 

The majority of studies indicate an inverse relationship between the severity of atopic dermatitis and vitamin D3 levels. Recently, in 2022, Ng and Yew published a systematic review and meta-analysis on the association between serum concentration of vitamin D3 and atopic dermatitis severity [71]. Based on the analysis of 20 studies with virtually 2000 cases of atopic dermatitis in humans, we concluded that lower serum concentration of vitamin D3 was associated with more severe atopic dermatitis, thus vitamin D supplementation could help to control the severity of clinical signs of atopic dermatitis. However, further research on the efficacy and optimal dosage of vitamin D is still needed [71]. Previous studies present the same conclusions that vitamin D supplementation is linked to a clinically relevant reduction in the severity of clinical symptoms in both adult and pediatric patients suffering from atopic dermatitis [72,73,74,75]. Similarly, in atopic dogs, an oral vitamin D supplementation decreases pruritus and skin lesions [76]. On the other hand, vitamin D3 as a highly potent hormone may lead to severe adverse effects, such as hypercalcaemia, hyperphosphatemia, and disseminated tissue calcification. In dogs, dietary vitamin D overdose may cause hypertension and nephropathy [77]. Therefore, to minimize the adverse effects, multiple VDR analogues were developed. VDR agonists, such as paricalcitol are not only less toxic, but also strongly stimulate AMPs in keratinocytes and other skin cells in vitro. In humans, they seem to be safer than cholecalciferol. Paricalcitol is the second-generation VDR activator, a synthetically manufactured analog of calcitriol, which is an active form of vitamin D. It is a VDR agonist with a potent immunomodulatory effect used in humans [78]. It showed a low potential to induce hypercalcemia and hyperphosphatemia [79]. Paricalcitol is considered for use in dogs. However, after the administration of this analogue to dogs Klinger et al. (2018) observed hypercalcemia in half of the tested animals [76]. Thus, we concluded that cholecalciferol was a better treatment option in dogs than paricalcitol. Moreover, they observed higher activity of vitamin D3 against lesions and pruritus in atopic dogs. In other studies, serious adverse effects in dogs have only been described in rare cases of accidental ingestion of high doses of vitamin D analogues, such as calcipotriol, calcitriol, and tocalcitol [80,81,82]. During proper treatment, clinicians observed some adverse effects, like erythema, skin irritation, or rarely photosensitivity; however, there is no data on the incidence of these complications in dogs [82].

In conclusion, low pre-existing calcitriol levels may predispose to the development of CAD. Moreover, Vitamin D3 improves the skin barrier defense, which reduces secondary skin infections. Therefore, vitamin D3 may turn out as an adjunctive therapy in CAD.

## Data Availability

Not applicable.

## References

[1] Hensel P., Santoro D., Favrot C., Hill P., Griffin C. (2015). Canine atopic dermatitis: Detailed guidelines for diagnosis and allergen identification. BMC Vet. Res..

[2] Santoro D., Marsella R., Pucheu-Haston C.M., Eisenschenk M.N.C., Nuttall T., Bizikova P. (2015). Review: Pathogenesis of canine atopic dermatitis: Skin barrier and host-microorganism interaction. Vet. Dermatol..

[3] Gedon N.K.Y., Mueller R.S. (2018). Atopic dermatitis in cats and dogs: A difficult disease for animals and owners. Clin. Transl. Allergy.

[4] Wollenberg A., Thomsen S.F., Lacour J.P., Jaumont X., Lazarewicz S. (2021). Targeting immunoglobulin E in atopic dermatitis: A review of the existing evidence. World Allergy Organ J..

[5] Nuttall T.J., Knight P.A., McAleese S.M., Lamb J.R., Hill P.B. (2002). Expression of Th1, Th2 and immunosuppressive cytokine gene transcripts in canine atopic dermatitis. Clin. Exp. Allergy.

[6] Gonzales A.J., Humphrey W.R., Messamore J.E., Fleck T.J., Fici G.J., Shelly J.A., Teel J.F., Bammert G.F., Dunham S.A., Fuller T.E. (2013). Interleukin-31: Its role in canine pruritus and naturally occurring canine atopic dermatitis. Vet. Dermatol..

[7] Leung D.Y.M. (2000). Atopic dermatitis: New insights and opportunities for therapeutic intervention. J. Allergy Clin. Immunol..

[8] Sinke J.D., Rutten V.P.M.G., Willemse T. (2002). Immune dysregulation in atopic dermatitis. Vet. Immunol. Immunopathol..

[9] Majewska A., Gajewska M., Dembele K., Maciejewski H., Prostek A., Jank M. (2016). Lymphocytic, cytokine and transcriptomic profiles in peripheral blood of dogs with atopic dermatitis. BMC Vet. Res..

[10] Bao L., Chau C., Bao J., Tsoukas M.M., Chan L.S. (2018). IL-4 dysregulates microRNAs involved in inflammation, angiogenesis and apoptosis in epidermal keratinocytes. Microbiol. Immunol..

[11] Santoro D. (2019). Therapies in canine atopic dermatitis: An update. Vet. Clin. Small Anim..

[12] Jaeger K., Linek M., Power H.T., Bettenay S.V., Zabel S., Rosychuk R.A., Mueller R.S. (2010). Breed and site predispositions of dogs with atopic dermatitis: A comparison of five locations in three continents. Vet. Dermatol..

[13] Favrot C. Clinical signs and diagnosis of canine atopic dermatitis. Proceedings of the 3 Congresso Latinoamericano de Dermatologia Veterinaria.

[14] Nuttall T.J., Marsella R., Rosenbaum M.R., Gonzales A.J., Fadok V.A. (2019). Update on pathogenesis, diagnosis, and treatment of atopic dermatitis in dogs. J. Am. Vet. Med. Assoc..

[15] Outerbridge C.A., Jordan T.J.M. (2021). Current knowledge on canine atopic dermatitis: Pathogenesis and treatment. Adv. Small Anim. Care.

[16] Cook C.P., Scott D.W., Miller W.H., Kirker J.E., Cobb S.M. (2004). Treatment of canine atopic dermatitis with cetirizine, a second generation antihistamine: A single-blinded, placebo-controlled study. Can. Vet. J..

[17] Olivry T., DeBoer D.J., Favrot C., Jackson H.A., Mueller R.S., Nuttall T., Prélaud P. (2015). International Committee on Allergic Diseases of Animals. Treatment of canine atopic dermatitis: 2015 updated guidelines from the International Committee on Allergic Diseases of Animals (ICADA). BMC Vet. Res..

[18] Saridomichelakis M.N., Olivry T. (2016). An update on the treatment of canine atopic dermatitis. Vet. J..

[19] Marsella R. (2021). Atopic dermatitis in domestic animals: What our current understanding is and how this applies to clinical practice. Vet. Sci..

[20] Bruet V., Mosca M., Briand A., Bourdeau P., Pin D., Cochet-Faivre N., Cadiergues M.C. (2022). Clinical guidelines for the use of antipruritic drugs in the control of the most frequent pruritic skin diseases in dogs. Vet. Sci..

[21] Ramió-Lluch L., Brazís P., Ferrer L., Puigdemont A. (2020). Allergen-specific immunotherapy in dogs with atopic dermatitis: Is owner compliance the main success-limiting factor?. Vet. Rec..

[22] Gonzales A.J., Bowman J.W., Fici G.J., Zhang M., Mann D.W., Mitton-Fry M. (2014). Oclacitinib (Apoquel®) is a novel Janus kinase inhibitor with activity against cytokines involved in allergy. J. Vet. Pharmacol. Ther..

[23] Cosgrove S.B., Wren J.A., Cleaver D.M., Walsh K.F., Follis S.I., King V.I., Tena J.K., Stegemann M.R. (2013). A blinded, randomized, placebo-controlled trial of the efficacy and safety of the Janus kinase inhibitor oclacitinib (Apoquel®) in client-owned dogs with atopic dermatitis. Vet. Dermatol..

[24] Little P.R., King V.L., Davis K.R., Cosgrove S.B., Stegemann M.R. (2015). A blinded, randomized clinical trial comparing the efficacy and safety of oclacitinib and ciclosporin for the control of atopic dermatitis in client-owned dogs. Vet. Dermatol..

[25] Panteri A., Strehlau G., Helbig R., Prost C., Doucette K. (2016). Repeated oral dose tolerance in dogs treated concomitantly with ciclosporin and oclacitinib for three weeks. Vet. Dermatol..

[26] Brussel L.V., Moyaert H., Escalada M., Mahabir S.P., Stegemann M.R. (2021). A masked, randomised clinical trial evaluating the efficacy and safety of lokivetmab compared to saline control in client-owned dogs with allergic dermatitis. Vet. Dermatol..

[27] Moyaert H., Van Brussel L., Borowski S., Escalada M., Mahabir S.P., Walters R.R., Stegemann M.R. (2017). A blinded, randomized clinical trial evaluating the efficacy and safety of lokivetmab compared to ciclosporin in client-owned dogs with atopic dermatitis. Vet. Dermatol..

[28] Lee S., Yun T., Koo Y., Chae Y., Lee D., Choi D., Choi Y., Kim H., Yang M.P., Kang B.T. (2021). Clinical efficacy of oclacitinib and lokivetmab in dogs with canine atopic dermatitis. J. Vet. Clin..

[29] Fazakerley J., Nuttall T., Sales D., Schmidt V., Carter S.D., Hart C.A., McEwan N.A. (2009). Staphylococcal colonization of mucosal and lesional skin sites in atopic and healthy dogs. Vet. Dermatol..

[30] Rodrigues-Hoffmann A., Patterson A.P., Diesel A., Lawhon S.D., Ly H.J., Stephenson C.E., Mansell J., Steiner J.M., Dowd S.E., Olivry T. (2014). The skin microbiome in healthy and allergic dogs. PLoS ONE.

[31] Meason-Smith C., Diesel A., Patterson A.P., Older C.E., Mansell J.M., Suchodolski J.S., Rodrigues-Hoffmann A. (2015). What is living on your dog’s skin? Characterization of the canine cutaneous mycobiota and fungal dysbiosis in canine allergic dermatitis. FEMS Microbiol. Ecol..

[32] Pierezan F., Olivry T., Paps J.S., Lawhon S.D., Wu J., Steiner J.M., Suchodolski J.S., Hoffmann A.R. (2016). The skin microbiome in allergen-induced canine atopic dermatitis. Vet. Dermatol..

[33] Torres S., Clayton J.B., Danzeisen J.L., Ward T., Huang H., Knights D., Johnson T.J. (2017). Diverse bacterial communities exist on canine skin and are impacted by cohabitation and time. Peer J..

[34] Sakamoto M., Asahina R., Kamishina H., Maeda S. (2016). Transcription of thymic stromal lymphopoietin via Toll-like receptor 2 in canine keratinocytes: A possible association of *Staphylococcus* spp. in the deterioration of allergic inflammation in canine atopic dermatitis. Vet. Dermatol..

[35] Bradley C.W., Morris D.O., Rankin S.C., Cain C.L., Misic A.M., Houser T., Mauldin E.A., Grice E.A. (2016). Longitudinal evaluation of the skin microbiome and association with microenvironment and treatment in canine atopic dermatitis. J. Investig. Dermatol..

[36] Older C.E., Rodrigues-Hoffmann A., Hoover K., Banovic F. (2020). Characterization of cutaneous bacterial microbiota from superficial pyoderma forms in atopic dogs. Pathogens.

[37] Sofou E.I., Aleksandrova S., Badulescu E., Chatzis M., Saridomichelakis M. (2022). Efficacy of antimicrobial treatment in dogs with atopic dermatitis: An observational study. Vet. Sci..

[38] Farver K., Morris D.O., Shofer F., Esch B. (2005). Humoral measurement of type-1 hypersensitivity reactions to a commercial *Malassezia* allergen. Vet. Dermatol..

[39] van Damme C.M.M., Willemse T., van Dijk A., Haagsman H.P., Veldhuizen E.J.A. (2009). Altered cutaneous expression of β-defensins in dogs with atopic dermatitis. Mol. Imm..

[40] Coates M., Blanchard S., MacLeod A.S. (2018). Innate antimicrobial immunity in the skin: A protective barrier against bacteria, viruses, and fungi. PLoS Pathog..

[41] Schauber J., Gallo R.L. (2008). Antimicrobial peptides and the skin immune defense system. J. Allergy Clin. Immunol..

[42] Santoro D., Bunick D., Graves T.K., Segre M. (2013). Evaluation of canine antimicrobial peptides in infected and noninfected chronic atopic skin. Vet. Dermatol..

[43] Leonard B.C., Affolter V.K., Bevins C.L. (2012). Antimicrobial peptides: Agents of border protection for companion animals. Vet. Dermatol..

[44] Santoro D. (2018). Evaluation of the secretion of antimicrobial peptides and antimicrobial effect of skin wash in atopic and healthy dogs: A preliminary study. Vet. Dermatol..

[45] Ganz T. (2003). Defensins: Antimicrobial peptides of innate immunity. Nat. Rev. Immunol..

[46] Zhao L., Lu W. (2014). Defensins in innate immunity. Curr. Opin. Hematol..

[47] Patil A.A., Cai Y., Sang Y., Blecha F., Zhang G. (2005). Cross-species analysis of the mammalian beta-defensin gene family: Presence of syntenic gene clusters and preferential expression in the male reproductive tract. Physiol. Genom..

[48] Yu J., Mookherjee N., Wee K., Bowdish D.M., Pistolic J., Li Y., Rehaume L., Hancock R.E. (2007). Host defense peptide LL-37, in synergy with inflammatory mediator IL-1beta, augments immune responses by multiple pathways. J. Immunol..

[49] Frohm M., Agerberth B., Ahangari G., Stâhle-Bäckdahl M., Lidén S., Wigzell H., Gudmundsson G.H. (1997). The expression of the gene coding for the antibacterial peptide LL-37 is induced in human keratinocytes during inflammatory disorders. J. Biol. Chem..

[50] Ong P.Y., Ohtake T., Brandt C., Strickland I., Boguniewicz M., Ganz T., Gallo R.L., Leung D.Y. (2002). Endogenous antimicrobial peptides and skin infections in atopic dermatitis. N. Engl. J. Med..

[51] Harder J., Bartels J., Christophers E., Schröder J.M. (2001). Isolation and characterization of human β-defensin-3, a novel human inducible peptide antibiotic. J. Biol. Chem..

[52] Maeda S., Fujiwara S., Omori K., Kawano K., Kurata K., Masuda K., Ohno K., Tsujimoto H. (2002). Lesional expression of thymus and activation-regulated chemokine in canine atopic dermatitis. Vet. Immunol. Immunopathol..

[53] Nuttall T.J., Knight P.A., McAleese S.M., Lamb J.R., Hill P.B. (2002). T-helper 1, T-helper 2 and immunosuppressive cytokines in canine atopic dermatitis. Vet. Immunol. Immunopathol..

[54] Leonard B.C., Marks S.L., Outerbridge C.A., Affolter V.K., Kananurak A., Young A., Moore P.F., Bannasch D.L., Bevins C.L. (2012). Activity, expression and genetic variation of canine β-defensin 103: A multifunctional antimicrobial peptide in the skin of domestic dogs. J. Innate Immun..

[55] Wang T.T., Nestel F., Bourdeau V., Nagai Y., Wang Q., Liao J., Tavera-Mendoza L., Lin R., Hanrahan J.W., Mader S. (2004). Cutting edge: 1,25-dihydroxyvitamin D3 is a direct inducer of antimicrobial peptide gene expression. J. Immunol..

[56] Holick M.F. (2007). Vitamin D deficiency. N. Engl. J. Med..

[57] Kechichian E., Ezzedine K. (2018). Vitamin D and the skin: An update for dermatologists. Am. J. Clin. Dermatol..

[58] Gross K.L., Wedekind K.J., Cowell C.S., Schoenherr W.D., Jewell D.E., Zicker S.C., Debraekeleer J., Frey R.A., Hand M.S., Thatcher C.D., Remillard R.L., Roudebush P. (2000). Nutrients. Small Animal Clinical Nutrition.

[59] Miller J., Gallo R.L. (2010). Vitamin D and innate immunity. Dermatol. Ther..

[60] White J.H. (2022). Emerging roles of vitamin D-induced antimicrobial peptides in antiviral innate immunity. Nutrients.

[61] van Etten E., Mathieu C. (2005). Immunoregulation by 1,25-dihydroxyvitamin D3: Basic concepts. J. Steroid Biochem. Mol. Biol..

[62] Oberg F., Botling J., Nilsson K. (1993). Functional antagonism between vitamin D3 and retinoic acid in the regulation of CD14 and CD23 expression during monocytic differentiation of U-937 cells. J. Immunol..

[63] Schauber J., Dorschner R., Coda A., Buchau A., Liu P., Kiken D., Helfrich Y., Kang S., Elalieh H., Steinmeyer A. (2007). Injury enhances TLR2 function and antimicrobial peptide expression through a vitamin D-dependent mechanism. J. Clin. Investig..

[64] Wang T.T., Dabbas B., Laperriere D., Bitton A.J., Soualhine H., Tavera-Mendoza L.E., Dionne S., Servant M.J., Bitton A., Seidman E.G. (2010). Direct and indirect induction by 1,25-Dihydroxyvitamin D3 of the NOD2/CARD15-defensin β2 innate immune pathway defective in Crohn Disease. J. Biol. Chem..

[65] Verway M., Bouttier M., Wang T.T., Carrier M., Calderon M., An B.S., Devemy E., McIntosh F., Divangahi M., Behr M.A. (2013). Vitamin D induces interleukin-1β expression: Paracrine macrophage epithelial signaling controls *M. tuberculosis* infection. PLoS Pathog..

[66] Gombart A.F., Borregaard N., Koeffler H.P. (2005). Human cathelicidin antimicrobial peptide (CAMP) gene is a direct target of the vitamin D receptor and is strongly up-regulated in myeloid cells by 1,25-dihydroxyvitamin D3. FASEB J..

[67] Schauber J., Dorschner R.A., Yamasaki K., Brouha B., Gallo R.L. (2006). Control of the innate epithelial antimicrobial response is cell-type specific and dependent on relevant microenvironmental stimuli. Immunology.

[68] Segaert S. (2008). Vitamin D regulation of cathelicidin in the skin: Toward a renaissance of vitamin D in dermatology?. J. Investig. Dermatol..

[69] Liu P.T., Stenger S., Tang D.H., Modlin R.L. (2007). Cutting edge: Vitamin D-mediated human antimicrobial activity against *Mycobacterium tuberculosis* is dependent on the induction of cathelicidin. J. Immunol..

[70] Liu P.T., Stenger S., Li H., Wenzel L., Tan B.H., Krutzik S.R., Ochoa M.T., Schauber J., Wu K., Meinken C. (2006). Toll-like receptor triggering of a vitamin D-mediated human antimicrobial response. Science.

[71] Ng J.C., Yew Y.W. (2022). Effect of vitamin D serum levels and supplementation on atopic dermatitis: A systematic review and meta-analysis. Am. J. Clin. Dermatol..

[72] Lee S.A., Hong S., Kim H.J., Lee S.H., Yum H.Y. (2013). Correlation between serum vitamin D level and the severity of atopic dermatitis associated with food sensitization. Allergy Asthma Immunol. Res..

[73] Dogru M. (2018). Is vitamin D level associated with the natural course of atopic dermatitis?. Allergol. Immunopathol..

[74] Hattangdi-Haridas S.R., Lanham-New S.A., Wong W.H.S., Ho M.H.K., Darling A.L. (2019). Vitamin D deficiency and effects of vitamin D supplementation on disease severity in patients with atopic dermatitis: A systematic review and meta-analysis in adults and children. Nutrients.

[75] Mansour N.O., Mohamed A.A., Hussein M., Eldemiry E., Daifalla A., Hassanin S., Nassar N., Ghaith D., Salah E.M. (2020). The impact of vitamin D supplementation as an adjuvant therapy on clinical outcomes in patients with severe atopic dermatitis: A randomized controlled trial. Pharmacol. Res. Perspect..

[76] Klinger C.J., Hobi S., Johansen C., Koch H.J., Weber K., Mueller R.S. (2018). Vitamin D shows in vivo efficacy in a placebo-controlled, double-blinded, randomised clinical trial on canine atopic dermatitis. Vet. Rec..

[77] Mellanby R.J., Mee A.P., Berry J.L., Herrtage M.E. (2005). Hypercalcaemia in two dogs caused by excessive dietary supplementation of vitamin D. JSAP.

[78] Sochorová K., Budinský V., Rozková D., Tobiasová Z., Dusilová-Sulková S., Spísek R., Bartůnková J. (2009). Paricalcitol (19-nor-1,25-dihydroxyvitamin D2) and calcitriol (1,25-dihydroxyvitamin D3) exert potent immunomodulatory effects on dendritic cells and inhibit induction of antigen-specific T cells. Clin. Immunol..

[79] Goldenberg M.M. (1999). Paricalcitol, a new agent for the management of secondary hyperparathyroidism in patients undergoing chronic renal dialysis. Clin. Ther..

[80] Fan T.M., Simpson K.W., Trasti S., Birnbaum N., Center S.A., Yeager A. (1998). Calcipotriol toxicity in a dog. JSAP.

[81] Hilbe M., Sydler T., Fischer L., Naegeli H. (2000). Metastatic calcification in a dog attributable to ingestion of a tacalcitol ointment. Vet. Pathol..

[82] Ho B., Ellison J., Edwards N., Bates N. (2021). Prevalence of vitamin D analogue toxicity in dogs. Clin. Exp. Dermatol..

